# From sounds to sentences: A longitudinal study of phonological and emergent literacy predictors of early writing in Turkish children

**DOI:** 10.1371/journal.pone.0361638

**Published:** 2026-10-26

**Authors:** Aysel Ferah Özcan, Hilal İlknur Tunçeli

**Affiliations:** 1 Primary Education Department, Faculty of Education, Sakarya University, Sakarya, Türkiye; 2 Early Childhood Education Department, Faculty of Education, Sakarya University, Sakarya, Türkiye; Macquarie University, AUSTRALIA

## Abstract

This longitudinal study examines how preschool children’s phonological awareness and emergent literacy skills relate to their early writing performance in first grade. Conducted within the context of a transparent Turkish orthography, the study aims to identify early cognitive-linguistic correlates of subsequent writing development. A total of 97 children (aged 66–77 months) from preschools in a socioeconomically diverse Turkish district were followed over two academic years. In the first year, participants were assessed using the LAP-3 Early Literacy Subtest and the Phonological Awareness Test to measure their emergent literacy and phonological skills. One year later, their writing performance was evaluated using a validated sentence construction assessment. Non-parametric statistical tests and correlation analyses were conducted to examine developmental variations and relationships among the variables. Results revealed statistically significant correlations between early writing performance and both phonological awareness (r = .46, p < .01) and emergent literacy (r = .32, p < .01). Specific sub-skills such as syllable segmentation, rhyming awareness, and letter-sound correspondence showed the strongest associations with writing performance. These skills did not differ significantly by age or gender. The findings highlight the foundational role of preschool phonological and emergent literacy skills in early writing development. This study contributes empirical data from a transparent orthography. Given the correlational design, these findings should be interpreted as evidence consistent with developmental literacy theories rather than as confirmation of causal or predictive mechanisms; they emphasize the importance of early identification and instructional support in preschool settings to promote future writing success.

## Introduction

Early literacy development plays a critical role in academic success and social integration throughout life, as emphasized by recent international reports [1,2]. Children who begin primary school with strong phonological awareness (PA) and print-related skills demonstrate significantly better reading and writing outcomes in later years, as shown in the Progress in International Reading Literacy Study (PIRLS) [3]. These findings highlight the importance of identifying early predictors of literacy, particularly in transparent orthographies such as Turkish, where phoneme-grapheme correspondences are highly consistent but have received less research attention compared to opaque languages like English or French [4].

This study is grounded in theoretical frameworks of emergent literacy [5] and the developmental progression of phonological awareness [6]. These models underscore the significance of oral language processing, phoneme manipulation, and letter-sound knowledge in the development of early writing skills. While most prior research has focused on decoding and reading fluency, relatively few studies have examined the longitudinal relationship between preschool phonological awareness and subsequent writing development, especially in non-English and transparent language contexts.

Focusing on the Turkish language, this study investigates the longitudinal associations between early writing skills in first grade and emergent literacy skills during preschool. Writing acquisition is a complex process involving both linguistic and cognitive components; thus, understanding its early predictors is essential for informing instructional practices and early intervention programs. According to Teale and Sulzby [5] literacy development begins at birth and progresses along a continuum toward conventional literacy. Research suggests that literacy evolves within broad social environments [6] and foundational skills such as print awareness, phonological awareness, and oral language are essential for both reading and writing [7].

Emergent literacy theory conceptualizes literacy development as a continuous process beginning at birth, in which young children construct knowledge about print, oral language, and symbolic representation well before formal reading instruction [5,8]. Within this framework, PA—the meta-linguistic ability to detect and manipulate the sound structure of spoken language independently of meaning—is one of several interrelated foundational components, alongside print awareness, alphabet knowledge, and oral language, that jointly support literacy acquisition [6,9–12]. Although PA is often treated as a component nested within the broader emergent literacy construct, the present study follows prior work that examines PA as a conceptually distinct predictor because of its specificity: PA indexes an auditory-metalinguistic skill that can, in principle, be assessed independently of print exposure, whereas emergent literacy as operationalized here (via the LAP-3 Early Literacy Subtest) primarily captures print-referenced and symbolic-production skills, such as letter and numeral writing and symbol copying. This conceptual separation allows the relative contributions of auditory-phonological versus print-referenced skills to be examined empirically rather than assumed a priori.

Much of the empirical evidence linking PA to literacy and writing outcomes originates from opaque orthographies such as English, where inconsistent grapheme-phoneme correspondences place heavier demands on phonological decoding and where PA effects on reading and spelling tend to be large and persistent [4,6,10–12]. In shallow, transparent orthographies such as Turkish, grapheme-phoneme mapping is highly consistent, and cross-linguistic evidence suggests that basic decoding and encoding skills are mastered comparatively quickly, which could either reduce the long-term predictive weight of PA relative to opaque languages or, alternatively, leave PA equally central because transparent mapping itself depends on accurate phonological segmentation [4]. Whether the associations typically reported between PA and writing in opaque orthographies generalize in magnitude to a transparent orthography such as Turkish, or whether orthographic transparency attenuates or strengthens this relationship, therefore remains an open empirical question and is a central motivation for the present study.

Early writing involves the development of letter and word recognition, orthographic accuracy, and morphological awareness, forming a base for more advanced literacy skills. Children begin by applying letter-sound correspondences and later engage in morphological and lexical analysis at the word level. By the end of first grade, phonological processing often gives way to more lexical and orthographic strategies [13]. Early literacy skills are strong predictors of later success in reading fluency, comprehension, spelling, and writing. Acquiring skills such as phoneme manipulation, word segmentation, grapheme-phoneme mapping, and vocabulary prior to formal schooling supports long-term reading and writing achievement [14].

These skills not only predict academic outcomes but also support broader linguistic fluency and cognitive development, including vocabulary acquisition and critical thinking [15,16]. Children who develop an understanding of written language and word structure before formal education are better prepared to apply these skills in literacy tasks. Such abilities have been shown to predict future reading competence [17,18] as they enhance phoneme-grapheme correspondence and simultaneous letter recognition.

Phonological awareness, a meta-linguistic skill, involves recognizing and manipulating the sound structure of language at various levels—from words and syllables to phonemes [9]. Mastery of phonemic processing and linguistic analysis is essential for literacy acquisition [10]. Emphasizing auditory processing over semantics, phonological awareness requires the ability to attend to and manipulate sounds in spoken language [11]. Children typically progress from manipulating syllables, to onsets and rimes, and eventually to individual phonemes [10–12].

Developmental theory suggests that phonological awareness comprises increasingly complex tasks depending on sound unit size and type of manipulation [19]. Tasks involving phoneme discrimination are more challenging than those involving word or syllable-level tasks [6]. While extensive research has been conducted on reading development, much less attention has been given to the development of spelling and writing skills in preschool-aged children. Moreover, existing research tends to focus on high-level writing processes such as text planning and grammar, leaving the foundational cognitive components of writing underexplored—namely, the phonological, print-referenced, and symbolic-production skills (e.g., phoneme manipulation, letter-sound knowledge, letter and numeral writing) that precede and scaffold conventional spelling.

This research gap may stem from the common perception that reading and writing rely on similar cognitive processes. However, emerging evidence suggests that reading and spelling/writing rely on both shared and distinct cognitive mechanisms, highlighting the need to investigate the specific developmental pathways of early writing. Recent studies have examined letter-name knowledge and letter writing in relation to spelling, but there remains limited understanding of how writing relates to broader early literacy and oral language skills in transparent languages such as Turkish.

While phonological awareness (PA) is universally acknowledged as the single most robust predictor of literacy acquisition in alphabetic languages, its specific role has been less studied in transparent orthographies like Turkish. Orthographic depth—the consistency of phoneme-grapheme mapping—is known to modulate the nature and timing of literacy skill development. Therefore, the primary motivation of this study is to test the universality of these foundational literacy mechanisms and determine if the high consistency of phoneme-grapheme mapping in Turkish alters the magnitude or nature of the predictive relationship between PA and early writing skills compared to studies conducted in opaque languages (e.g., English). By examining this relationship in Turkish, we aim to provide empirical data to refine and reinforce developmental literacy theories across different orthographic systems.

Given the substantial number of children worldwide who fail to achieve literacy proficiency, and the foundational role of early childhood in establishing these skills, it is essential to investigate the links between emergent literacy and early writing. To date, these relationships have not been adequately examined in Turkish, a transparent orthography. This study aims to address this gap and contribute original insights into how early cognitive-linguistic skills shape writing development within a longitudinal framework.

Based on the theoretical frameworks of emergent literacy and phonological awareness, as well as prior empirical findings, the following hypotheses were formulated:

H1: Preschool children’s phonological awareness skills will be significantly and positively associated with their early writing performance in the first grade.
H2: Preschool children’s emergent literacy skills (as measured by the LAP-3 Early Literacy Subtest) will be significantly and positively associated with their early writing performance in the first grade.
H3: Phonological awareness will show a stronger association with early writing performance than emergent literacy components.
H4 (secondary comparison): Building on prior mixed evidence regarding fine-motor and self-regulatory advantages favoring girls in early writing-related tasks and age-graded maturation in phonological processing [6,19], age and gender were examined as descriptive/control comparisons rather than central theoretical predictions. We anticipated no statistically significant differences in phonological awareness, emergent literacy, or early writing performance based on gender or age within the narrow developmental range studied, given that range restriction limits the detectable magnitude of such differences.

### Purpose of the study

The primary aim of this study is to determine the relationship between preschool children’s emergent literacy and phonological awareness skills and their early writing performance in the first grade. The following research questions operationalize Hypotheses H1-H4 above and guided the descriptive, comparative, and correlational analyses reported below:

1. What are the levels of phonological awareness and emergent literacy among preschool children?
2. What are the early writing skill levels of these children one year later?
3. Do children’s levels of phonological awareness, emergent literacy, and early writing (sentence dictation task) differ significantly by gender and age?
4. Is there a statistically significant relationship between children’s phonological awareness, emergent literacy, and early writing skills (sentence dictation task)?

## Method

This study employed a descriptive research design, specifically a panel-type longitudinal approach within a quantitative research framework. Longitudinal research allows for the examination of developmental changes and temporal patterns by collecting data from the same individuals over multiple time points [20]. A panel design is well-suited, in principle, for identifying developmental trajectories and potential causal relationships when paired with regression or structural modeling; the present study, however, used the panel design to collect variables at two time points and examined their associations using correlational and non-parametric methods rather than regression modeling, so the design’s full potential for trajectory- or causality-focused inference was not realized here. This choice still aligns with the study’s core objective of examining how preschool emergent literacy and phonological awareness relate to writing abilities in first grade, while leaving stronger developmental or causal claims to future work using regression-based approaches.

Despite practical challenges such as participant attrition and the need for sustained data collection, longitudinal studies are highly valued in educational research for their ability to produce robust, time-sensitive findings. Following the same cohort of preschool children into the first grade provides richer insights into how early cognitive-linguistic skills influence subsequent writing performance (sentence dictation task). Accordingly, the selected approach enhances both the validity and interpretability of the study’s results within the field of developmental literacy research.

### Study group

The study was conducted in preschool classrooms (referred to in the Turkish system as “anaokulu”/kindergarten, serving children aged approximately 36–72 months) in a district center situated at a considerable distance from one of Türkiye’s major cities. In this context, “preschool” and “kindergarten” are used interchangeably to denote the same non-mandatory, pre-primary educational setting, which does not provide formal reading or writing instruction; such instruction begins only with first grade at age 66–72+ months. This district was selected due to its accessibility, logistical feasibility, and socioeconomic diversity, which allowed for more efficient data collection and the opportunity for year-to-year follow-up.

The district population is characterized by a mix of working-class, commercial, and agricultural households. Within the kindergarten programs in the district, children were divided into two cohorts: those scheduled to begin primary school in one year, and those set to begin in two years. Although over 200 children were enrolled in kindergartens in the district, attendance rates were relatively low, as kindergarten education in Turkey is not mandatory.

Initially, 180 children were identified as potential participants. Of these, 30 children under the age of 60 months were excluded. Consent forms were distributed to the families of the remaining 150 children, and 120 families provided voluntary written consent. During the first year, data were collected from 112 children, though 8 could not be assessed due to inconsistent school attendance. In the second year, 97 children were successfully re-contacted and included in the final study group, as some families had moved or changed contact information. The study was conducted with a cohort of N = 97 participants (first year 112, second year 97 children). While the sample size is small, we confirm it represented the maximum reachable cohort available for the longitudinal component of the study, given the recruitment constraints. Furthermore, the size was deemed statistically sufficient to perform the planned correlational and non-parametric comparison analyses, providing adequate power to detect the primary effects under investigation. Regarding attrition, the 15 children lost to follow-up between the first-year assessment (n = 112) and the first-grade assessment (n = 97) were, according to school and family records, not retained primarily because their families relocated or changed contact information between school years, rather than for reasons documented as related to the children’s academic performance. However, a formal statistical comparison of retained versus attrited children on first-year phonological awareness and LAP-3 scores was not conducted, as this comparison was not planned at the data-collection stage and the necessary records could not be reconstructed retrospectively. This is a limitation of the study, since family mobility can itself covary with socioeconomic circumstances, and the possibility of attrition-related bias cannot be excluded on the basis of the available records.

Based on the reachable population of 150, a sample size of 90 was considered statistically adequate. With 97 participants, the final study group was deemed sufficient for longitudinal analysis. The 51.5% of the study group consists of girls (n = 50) and 48.5% consists of boys (n = 47).

The study sample consisted of children recruited from various kindergartens within a Turkish district, resulting in an observed age range of 66–77+ months at the time of initial assessment. This broad age span is primarily attributed to the non-mandatory nature of kindergarten education in Turkey at the time, alongside varying parental decisions regarding the timing of school entry. As this was a longitudinal study focusing on children preparing for primary school, this age window was purposefully included to capture the range of typical developmental readiness. Furthermore, all assessments were conducted using Turkish-language standardized instruments. Consequently, the participants were implicitly Turkish-speaking. And language was not used as a separate variable for exclusion, as the study’s focus was on literacy development within the standard age window for Turkish primary school matriculation.

The distribution of the study group according to the age group variable indicate that 14.4% (n = 14) of the study group is 66–68 months old; 32% (n = 31) is 69–71 months old; 18.6% (n = 18) is 72–74 months old; 25.8% (n = 25) is 75–77 months old; and 9.3% (n = 9) consists of children aged 77 months and older.

A priori power analyses were conducted using G*Power (v.3.1) to determine the required sample size for detecting medium effect sizes (α = .05, 1 − β = .80) as defined by Cohen [21]. For Spearman’s rank-order correlations (ρ = .30), a minimum sample size of N = 85 was required, indicating that the final sample (N = 97) was sufficient for the primary correlational analyses.

In contrast, group comparison analyses required larger sample sizes (Mann–Whitney U: N = 128; Kruskal–Wallis: N = 159). Therefore, these analyses were relatively underpowered, and non-significant findings related to group differences were interpreted with caution due to the increased risk of Type II error.

Additionally, post-hoc power analysis for the observed correlation between phonological awareness and writing performance (r = .46) indicated very high statistical power (1 − β > .99), further supporting the adequacy of the sample size for the main research question.

### Data collection tools

In this study, three standardized instruments with established validity and reliability were employed for data collection: (1) the Phonological Awareness Form, a subtest of the Early Literacy Skills Test developed by Ergül et al. [22]; (2) the Early Literacy Subtest of the LAP-3, adapted into Turkish by Tunçeli [23] and registered with copyright in Turkey; and (3) the Initial Writing Skills Scale, developed by Ferah-Özcan [24]. During the preschool phase, participants were individually administered both the Phonological Awareness Form and the LAP-3 Early Literacy Subtest. Subsequently, in the second semester of the first grade (April), the Initial Writing Skills Scale was administered to assess their foundational writing abilities.

#### 1. LAP-3 Early Literacy Subtest.

The Early Literacy Subtest of the Learning Accomplishment Profile-3 (LAP-3) was utilized in this study to assess preschool children’s developmental status. The instrument was originally developed by Sanford [25] and later revised by Hardin and Peisner Feinberg [25], with subsequent studies supporting its validity and reliability. It was translated and adapted into Turkish by Tunçeli [23]. The LAP-3 covers a wide range of developmental domains—gross motor, fine motor, pre-writing, cognitive, language, self-help, and personal/social [25]—only the pre-writing domain was administered in the present study. In the original English-language LAP-3, this domain is formally titled “Pre-Writing,” not “early literacy.” In the Turkish adaptation used in this study, the same domain was translated and relabeled as “Okuma Yazmaya Hazırlık” (“Reading-Writing Readiness”) [23]; neither version includes a distinct prewriting subscale separate from this domain. The label “Early Literacy Subtest,” used throughout this manuscript, is a functional English-language description of the domain’s content (early print awareness and pre-writing/symbolic-production skills) rather than the official title used in either the original instrument or its Turkish adaptation. This subtest consists of 38 items designed to assess children’s reading and writing readiness skills and is hierarchically organized according to chronological age. Prior to administration, each child’s chronological age was calculated, and assessment began with age-appropriate items. Responses were scored dichotomously as correct (1) or incorrect (0).

In the Early Literacy Subtest of the LAP-3, children are given tasks such as copying letters and words, writing numerals, writing their first and last names, and copying symbols and shapes (e.g., a plus sign and a diamond shape). For example, for children aged 66–71 months, two items include writing their name (item no. 33) and writing numerals from 1 to 9 (item no. 35), whereas for children aged 72 months and older, two corresponding items include writing their first and last names (item no. 36) and writing numerals from 1 to 19 (item no. 37). Consistent with the dichotomous scoring convention used throughout the subtest, both the name-writing and numeral-writing items were scored as 1 only if every element (i.e., every letter of the name, or every numeral in the specified range) was written correctly, and as 0 if one or more errors occurred; this all-or-nothing criterion made a score of 1 comparatively difficult to obtain and is an intentional feature of the original LAP-3 scoring system rather than a deviation introduced in the Turkish adaptation. Age-based item variation in the LAP-3 Early Literacy Subtest is limited to these two production tasks (name writing and numeral writing); the visual-motor integration items (e.g., copying a plus sign or a diamond shape) and the early print-knowledge items (e.g., letter recognition, symbol matching) are identical across the age bands included in the present sample and were administered to all children using the same materials. Although numeral writing is not strictly linguistic, it is included as an indicator of symbolic representation and visual-motor coordination, which are foundational for early writing development.

Importantly, the reading-related items in the LAP-3 Early Literacy Subtest do not involve conventional reading of continuous text or sentence-level decoding. Instead, these items assess early print awareness and pre-reading skills, such as recognizing letters, identifying familiar written symbols, matching visual forms, and reproducing simple letter or word patterns. These tasks are designed to evaluate children’s emerging understanding of the symbolic nature of written language rather than their ability to read fluently. For conceptual clarity, the items in the Early Literacy Subtest can be grouped into four domains: (1) numeral writing (e.g., writing numbers 1–19), (2) name writing (e.g., writing own name and surname), (3) visual-motor integration (e.g., copying shapes such as a diamond or plus sign), and (4) early print knowledge (e.g., recognizing letters, copying letter forms, and matching visual symbols with their corresponding representations). The scoring procedure assigns one point for each correctly completed item (dichotomous scoring), yielding a maximum total score of 38. The total score reflects the overall mastery level across the subtest’s dimensions. In the present study, emergent literacy was operationalized as a multidimensional construct assessed through the Early Literacy Subtest of the LAP-3. This construct encompasses early print awareness, symbolic representation, and foundational writing-related skills. Specifically, it includes tasks related to name writing, numeral writing, recognition and reproduction of written symbols, and early print knowledge. While some items (e.g., shape copying) reflect visual–motor integration, these are considered supportive skills within early writing development rather than primary indicators of literacy. Of these four conceptual domains, only name writing and numeral writing require the child to actively produce written symbolic output (i.e., to write), the same response modality as the later sentence-dictation outcome measure; the visual-motor integration items (shape copying) and early print-knowledge items (letter/symbol recognition and matching) instead involve reproduction or recognition rather than free production. This conceptual proximity to the outcome measure was the basis for reporting name writing and numeral writing as separate rows in Tables 1 and 4, alongside the LAP-3 total score, rather than all four subdomains. This is a conceptually motivated rather than purely statistical selection; the visual-motor integration and print-knowledge subdomains were not reported at the item level, and a fuller item-level breakdown of all four subdomains remains a direction for future analysis of this dataset. Accordingly, all analyses are based on predefined subcomponents of the LAP-3, rather than selectively chosen items based on statistical outcomes.

The KR-20 internal consistency coefficient for this subscale by age was calculated as.90. The scale is administered individually to children and takes approximately 20 minutes per child. In this study, the full set of assessments was completed in approximately 40 hours (four weeks) for 112 children.

#### 2. Phonological Awareness Test.

A standardized and validated Phonological Awareness Test, a subcomponent of the Early Literacy Skills Test [22], was administered to evaluate the children’s phonological processing abilities. The test comprises a total of eight distinct subskills: rhyme awareness, initial/final sound matching, sentence/word segmentation, syllable blending, syllable segmentation, initial/final sound deletion, and sound manipulation

Each subtest carries a maximum score of 4 points, yielding a potential total maximum score of 32 points for the entire test. This test comprises several sub-tasks (e.g., rhyming, syllable blending, phoneme isolation) designed to assess children’s awareness of the sound structure of language. Each item is scored based on accuracy, typically assigning 1 point for a correct response. The scores across all sub-tasks are summed, resulting in a maximum total score of [*max score 32*]. Detailed scoring rubrics for individual subtests ensured consistency, particularly for tasks involving segmentation and blending. The composite score obtained from all subtests was used as a comprehensive variable representing the child’s general level of phonological awareness in this study. Exploratory and confirmatory factor analyses confirmed its construct validity (χ²/df = 1.14, RMSEA = .019, CFI = .99). Test scores were found to vary based on socioeconomic status, with effect sizes ranging between.05 and.14. As data were collected toward the end of the school term, no significant age-related differences were observed. The KR-20 internal consistency coefficient for the phonological awareness subscale was reported as.87. The test is widely used in Turkey for the individual identification of children with learning difficulties. The average administration time is approximately 20 minutes per child. In this study, implementation was completed in around 40 hours (approximately four weeks) for 112 children.

#### 3. Initial Writing Skills Scale (Sentence Dictation Task).

The Initial Writing Skills Scale, developed by Ferah-Özcan [24], was used to assess children’s foundational writing skills through a sentence dictation task administered individually to each child (i.e., one-on-one with the examiner, not in a group/classroom setting) during the second semester of first grade. The task consisted of three sentences of increasing complexity (comprising 4, 5, and 6 words, respectively), resulting in a total of 15 words.

Scoring focused strictly on the orthographic accuracy of each word produced by the child. Each written word was evaluated dichotomously: words written accurately and in compliance with Turkish orthographic conventions were scored as 1, whereas words containing spelling errors or resulting in unintelligibility were scored as 0. The final score was calculated as the percentage of correctly written words out of the total 15 words.

Although the original scale allows for more detailed evaluation approaches, including consideration of phonetic plausibility, no partial credit scoring was applied in the present study. Instead, a strict correct/incorrect scoring procedure was adopted to ensure consistency and clarity in data analysis.

The assessment procedure involved the administrator reading each sentence aloud twice, after which the child transcribed the sentence without visual cues. The estimated completion time was approximately 15 minutes. In this study, the sentence dictation task was administered over a one-month period (four weeks) in April.

It is important to note that scoring was limited to word-level orthographic accuracy and did not include a detailed analysis of additional writing features such as punctuation or capitalization, except insofar as they contributed to determining orthographic correctness. The scale demonstrated a unidimensional structure, as indicated by exploratory factor analysis. Sentence dictation task performance was calculated as the percentage of correctly written words out of the total number of target words.

The assessment procedure involved the administrator reading each sentence aloud twice, after which the child transcribed the sentence without visual cues. The estimated completion time was approximately 15 minutes. In this study, the sentence dictation task was administered over a one-month period (four weeks) in April, at the beginning of the second semester of first grade.

For the current study group, the internal consistency coefficient was calculated as.79 for the LAP-3 Early Literacy Subtest and.69 for the Phonological Awareness Form of the Early Literacy Skills Test. Given that these values are close to the commonly accepted reliability threshold of.70, the data obtained from these measures can be considered sufficiently reliable for research purposes.

### Procedure

Following the identification of the study group, the necessary permissions were obtained from the Provincial Directorate of National Education for the schools where the implementation would take place. Informed consent was obtained from the children’s parents. During the participant selection process, teachers and parents were consulted, and children with identified speech or writing difficulties or any diagnosed developmental concerns were excluded from the study. Additionally, based on teacher reports, all participating children were considered to demonstrate typical developmental and learning characteristics appropriate for their age group. After securing all required permissions, the implementation process was initiated. The data collection process was conducted between March 16, 2022 and June 7, 2024.

During the preschool phase, participants were individually administered the Phonological Awareness Form and the Early Literacy Subtest of the LAP-3. Subsequently, in the second semester of first grade (April), the Initial Writing Skills Scale was administered to assess students’ foundational writing abilities (sentence dictation task).

All analyses reported in this study are based solely on data obtained from these instruments. The specific LAP-3 sub-items included in the correlation analyses are presented in Table 4.

The Phonological Awareness Test required approximately 20 minutes per child. The LAP-3 Early Literacy Subtest was also individually administered and took about 20 minutes per child. The Initial Writing Skills Scale had an estimated completion time of approximately 15 minutes. In total, the full set of assessments was completed in approximately 40 hours over a four-week period with 112 children.

### Ethical statement

Ethical approval for this study was obtained from the Ethics Committee of [University Name] (Decision No: 05/40; March 9, 2022). Informed consent was obtained from all parents prior to participation.

### Data analysis

For the purpose of data analysis, skewness and kurtosis values were initially calculated to assess the normality of the distributions. The data related to the Phonological Awareness Form of the Early Literacy Skills Test and the Initial Writing Skills scores were found to follow a normal distribution (skewness and kurtosis ≤ ±1.5). In contrast, the LAP-3 Early Literacy Subtest scores deviated from normality, as indicated by skewness and kurtosis values exceeding ±1.5, as well as the results of the Shapiro–Wilk test (p ≤ .00).

Given the non-normal distribution of the LAP-3 subtest data, non-parametric statistical tests were employed. Specifically, the Mann–Whitney U test was used to compare differences by gender, and the Kruskal–Wallis test was applied to examine differences across age groups for the variables of emergent literacy, phonological awareness, and initial writing skills. While the Kruskal–Wallis test enables comparison across multiple independent groups, the Mann–Whitney U test is suitable for comparisons between two groups. The Kruskal–Wallis H test was employed to investigate differences across age groups. Participants were grouped into 2-month intervals (e.g., 66–67 months, 68–69 months) to reflect the available demographic data and the established developmental readiness cohorts used during their initial preschool enrollment and assessment. This grouping strategy was chosen to accurately represent the structure in which the data were originally collected. It should be noted that the use of relatively narrow age intervals within a limited developmental range (66–77 months) may have contributed to the non-significant age differences observed, as developmental changes within such short intervals may not be sufficiently pronounced to yield statistically significant differences [26].

To investigate the associations among emergent literacy, phonological awareness, and early writing performance, Spearman’s rank-order correlation coefficient was calculated. In addition to overall test scores, specific subcomponents of the LAP-3 Early Literacy Subtest—such as “Writing Name and Surname” and “Writing Numbers from 1 to 19”—were included in the correlation analysis due to their direct relevance to foundational literacy skills. The variables used in the correlation analysis were derived solely from the LAP-3 Early Literacy Subtest total score and its relevant sub-items, determined a priori based on the theoretical structure of the instruments rather than selected based on statistical outcomes (see Procedure, above, for the full list of sub-items).

Similarly, subdimensions of the Phonological Awareness Form—including rhyme awareness, initial and final sound matching, sentence and word segmentation, syllable blending, and initial and final sound deletion—were analyzed individually to explore their specific contributions to early writing development. This comprehensive approach aimed to identify which sub-skills were most closely associated with children’s initial writing abilities. Effect sizes were calculated (r and ε²) and interpreted according to Cohen [21]. Post-hoc power analyses were also conducted.

## Results

The following section presents the key findings of this longitudinal study, which examined the relationships between preschoolers’ early writing performance and their foundational cognitive-linguistic skills, including phonological awareness and emergent literacy competencies. Particular emphasis is placed on how these early abilities relate to writing outcomes in the first grade within the context of a transparent orthography. The analysis also explores potential variations based on demographic factors such as age and gender, as well as the differential associations of specific emergent literacy skills (e.g., name writing) with writing performance. These findings provide correlational evidence relevant to the developmental study of writing acquisition and carry potential implications for early literacy instruction. Descriptive statistics for all variables are presented in Table 1. All reported associations are based on predefined constructs determined a priori.

**Table 1:** Descriptive statistics for variables.

Variables	N	Min.	Max.	$\underset{―}{X}$	S	Skewness		Kurtosis	
						Statistic	Std. Error	Statistic	Std. Error
Phonological awareness total	97	3.00	31.00	18.78	5.15	−.69	.24	.88	.48
Early Literacy Subtest total	97	28	38	35.61	1.99	−1.63	.24	3.17	.48
Sentence dictation task	97	6.66	100	70.64	20.85	−.95	.24	.71	.48
Write his/her name and surname.	97	0	1	.11	.31	2.47	.24	4.22	.48
Write the numbers from 1 to 19.	97	0	1	.72	.45	−1.00	.24	−1.01	.48
Rhyme awareness	97	0	4	3.10	.88	−.94	.24	1.27	.48
Initial Sound Matching	97	0	4	2.72	1.11	−.39	.24	−.84	.48
Final Sound Matching	97	0	4	2.40	1.11	−.11	.24	−.90	.48
Sentence Segmentation	97	0	4	2.94	1.31	−1.01	.24	−.18	.48
Word Segmentation	97	0	4	3.40	1.18	−2.08	.24	3.23	.48
Syllable Blending.	97	0	4	3.31	1.34	−1.75	.24	1.49	.48
Initial Sound Deletion	97	0	3	.19	.65	3.73	.24	13.20	.48
Final Sound Deletion	97	0	3	.72	1.37	1.59	.24	.88	.48

Table 1 shows that the number of children participating in both the pre-reading and writing and phonemic awareness test applications is 97. The number of children reached in the first grade of primary school is also 97. Thus, the final study group for the second year was determined to be 97 individuals.

Since the scores of the variables were not normally distributed according to gender and age, and the sample size was small (Shapiro Wilk > .05), the Mann-Whitney U test was applied to determine whether the scores of phonological awareness and initial writing skills differed by gender. The obtained results are presented in Table 2.

**Table 2:** Mann-Whitney U test findings for variable differences by gender variable.

	n	S	$\underset{―}{X}$	Mean
Sentence Dictation Task	50	20.85	70.64	51.90
	47			45.91
	97			
Phonological Awareness	50	5.15	18.78	53.64
	47			44.06
	97			
LAP-3 Early Literacy Subtest	50	1.99	35.61	53.55
	47			44.16
	97			

p ≥ .05.

Gender differences in were examined using the Mann-Whitney U test, which compares the mean/median ranks of the dependent variable between two independent groups (boy and girl). Table 2 presents the results, correctly reporting the median ranks (under the ‘Mean’ column in the table format) for each gender group, along with the overall Mann-Whitney U test statistic. The inclusion of these median ranks ensures that the reader clearly sees the groups (Gender) for which the comparison was performed and the basis for the non-parametric test outcome.

According to Table 2, the LAP-3 literacy readiness test scores (Md girls = 53.55 n = 50, Md boys = 44.16 n = 47, U = 947.50, z = −1.696, p ≥ . 05, r = −0.17) phonemic awareness (Md girls = 53.64 n = 50, Md boys = 44.06 n = 47, U = 943.00, z = −1.679, p ≥ .05, r = −0.17) and sentence dictation task scores (Md girls = 52.08 n = 50, Md boys = 44.06 n = 47, U = 1030.00, z = −1.054, p ≥ . 05, r = −0.15) do not differ by gender (p ≥ . 05). For writing success, phonemic awareness, and LAP-3 reading readiness, the effect size has been determined to be low (Cohen criteria). 1 = small). All effect sizes were in the small range, indicating minimal practical gender-related differences. Statistical power was found to be low for analyses examining the effects of gender on phonological awareness (1 − β 0.35, low), early literacy (1 − β 0.35, low), and writing outcomes (1 − β 0.30, low). To determine whether phonological awareness and sentence dictation task scores differ by age, the Kruskal-Wallis test was applied.

The results are presented in Table 3.

**Table 3:** Kruskal Wallis test findings for the difference in dependent variables by age.

	N	S	$\underset{―}{X}$	Mean	Kruskal Wallis
Sentence Dictation Task	14	20.85	70.64	46.04	2.26
	31			49.42	
	18			50.58	
	25			49.72	
	9			47.00	
	97				
Phonological Awareness	14	5.15	18.78	42.36	3.11
	31			44.52	
	18			54.94	
	25			51.56	
	9			55.78	
	97				
LAP-3 Early Literacy Subtest	14	1.99	35.61	39.89	3.72
	31			45.35	
	18			53.31	
	25			52.86	
	9			56.39	
	97				

p ≥ .05.

To investigate age-related differences in the non-parametric Kruskal-Wallis test was employed. This test is specifically designed to compare the mean/median ranks of the dependent variable across three or more independent groups (the defined age groups). As shown in Table 3, the results correctly list the median ranks (under the ‘Mean’ column) for each age group, alongside the overall Kruskal-Wallis test statistic, explicitly indicating the groups (Age) that were compared in the analysis.

Table 3 shows that the variables did not differ significantly according to age groups (≥. 05). (For the variables, Group 1, n = 14, Group 2 n = 31, Group 3 n = 18, Group 4 n = 25, Group 5 n = 9). The median values presented by the groups show a slight increase with age but do not reveal a significant difference. Regarding sentence dictation task, the lowest median is exhibited by the group with relatively older or younger ages, such as 66–68 months and 75–77 months. This finding regarding sentence dictation task should be interpreted with caution. No statistically significant differences were observed across age groups for sentence dictation performance (H = 2.26, p ≥ .05, ε² = .02), phonological awareness (H = 3.11, p ≥ .05, ε² = .03), or emergent literacy (H = 3.72, p ≥ .05, ε² = .04). Effect sizes were small, suggesting that within this restricted age range, developmental differences were not sufficiently large to yield distinct performance levels. Statistical power was found to be low for analyses examining the effects of age on writing outcomes (1 − β 0.30, very low)

Table 4 presents the Spearman’s rank-order correlation coefficients examining the relationships among phonological awareness, emergent literacy, and early writing performance. The results indicate that the sentence dictation task was moderately and positively correlated with both phonological awareness total scores (r = .46, p < .01) and the LAP-3 Early Literacy Subtest total score (r = .32, p < .01), supporting H1 and H2. Because the association with phonological awareness was numerically larger than that with emergent literacy, this pattern is also consistent with H3. These results indicate that both phonological and emergent literacy skills are significantly associated with early writing performance.

**Table 4:** Spearman’s rank order correlation findings for the relationship between variables.

	Phonological Awareness Total	LAP-3 Early Literacy Total	Sentence Dictation Task	Write his/her name and surname.	Write the numbers from 1 to 19.	Rhyme awareness	Initial Sound Matching	Final Sound Matching	Sentence Segmentation	Word Segmentation	Syllable Blending.	Initial Sound Deletion
LAP-3 Early Literacy Total	.30^**^											
Sentence Dictation Task	.46^**^	.32^**^										
Write his/her name and surname.	.04		.20^**^									
Write the numbers from 1 to 19.	.30^**^		.35^**^									
Rhyme awareness	.37^**^	.13	.27^**^	.14^*^	.09							
Initial Sound Matching	.54^**^	−.05	.09	−.11	.06	.25^**^						
Final Sound Matching	.62^**^	.10	.23^**^	−.02	.12	.16	.33^**^					
Sentence Segmentation	.61^**^	.41^**^	.29^**^	.09	.37^**^	.11	.16^*^	.15				
Word Segmentation	.51^**^	.23^**^	.43^**^	.05	.14	.14	.13^*^	.28^**^	.31^**^			
Syllable Blending.	.50^**^	.15	.46^**^	.06	.13	.15	.13	.18	.27^*^	.48^**^		
Initial Sound Deletion	.34^**^	−.03	.24^**^	.11	−.03	.21^**^	.15^*^	.10	.13	.12	.04	
Final Sound Deletion	.53^**^	.20^**^	.18^*^	.04	.26^*^	−.01	.09	.22^*^	.28^**^	.15	.11	.30^**^

*p < 0.05, **p < 0.01.

When the subcomponents of the LAP-3 Early Literacy Subtest were examined in relation to external variables, writing numbers from 1 to 19 showed a moderate positive correlation with sentence dictation performance (r = .35, p < .01), whereas name writing demonstrated a weaker but still significant association (r = .20, p < .05). These findings indicate that specific emergent literacy skills contribute differentially to early writing development.

With respect to early writing performance, several phonological awareness subskills showed moderate positive correlations with the sentence dictation task, including syllable blending (r = .46, p < .01), word segmentation (r = .43, p < .01), and sentence segmentation (r = .29, p < .01). In addition, rhyme awareness (r = .27, p < .01) and final sound matching (r = .23, p < .05) were also significantly associated with writing performance, although at a lower magnitude. These findings indicate that both larger phonological units (e.g., syllables and words) and more fine-grained phonological processing skills contribute to early writing development.

Relationships among phonological awareness, emergent literacy, and early writing skills were examined using Spearman’s rank order correlation. Effect sizes were interpreted as small (ρ ≈ .10), moderate (ρ ≈ .30), and large (ρ ≥ .50). Statistical power was found to be very high for analyses examining the effects of phonological awareness on writing outcomes (1 − β = 0.99), high for analyses examining the effects of early literacy (LAP-3) on writing outcomes (1 − β = 0.93), and very high for analyses examining the effects of syllable blending on writing outcomes (1 − β = 0.99 and 1 − β = 0.98).

### Qualitative examples of writing performance

The strong correlation between phonological awareness and sentence dictation performance (r = .46) was visually reflected in the quality of the children’s written products. To illustrate the developmental stages captured by the scale, representative examples of dictated sentences from children scoring in the lower, middle, and upper quartiles of the phonological awareness distribution are provided below.

The child in Example 1 (Fig 1) can spell 15 words in the dictated sentences without errors, using Turkish spelling.

**Fig 1:**
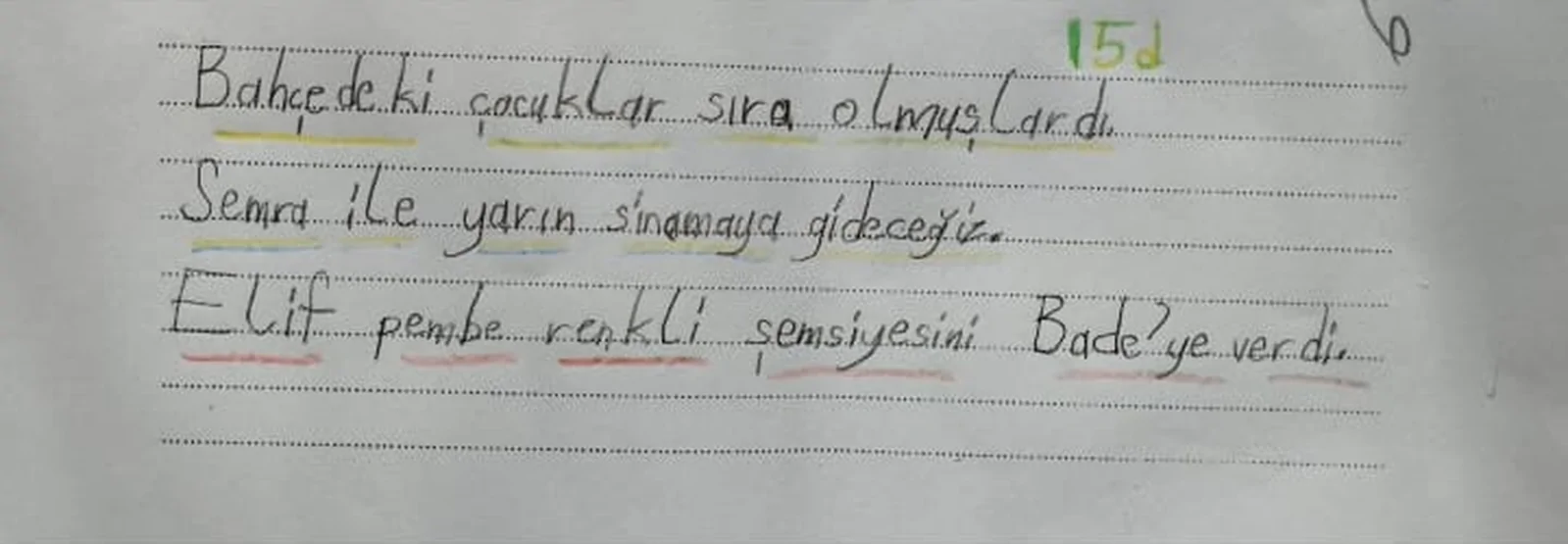
A student who can write in accordance with Turkish spelling.

The performance of Student Example 2 (Fig 2) indicates a struggle with **syllabification** (syllable segmentation). The student successfully wrote words for which they were able to establish letter-sound correspondence and segment the syllables. However, the student failed to correctly transcribe words that contain more syllables and require the use of Turkish suffixes, such as ‘Bahçedeki,’ ‘gideceğiz,’ and ‘şemsiyesini.’ It is observed that while the word ‘Bade’ye’ was produced mechanically (in terms of letter sequence), it was not written in compliance with Turkish orthographic convention (e.g., regarding the use of the apostrophe with proper nouns). Furthermore, the student substituted the dictated word ‘olmuşlardı’ with a non-dictated, invented form, ‘halindey,’ suggesting a breakdown in processing complex phonological structures. Overall, only 7 out of 15 words were written in compliance with Turkish orthography.

**Fig 2:**
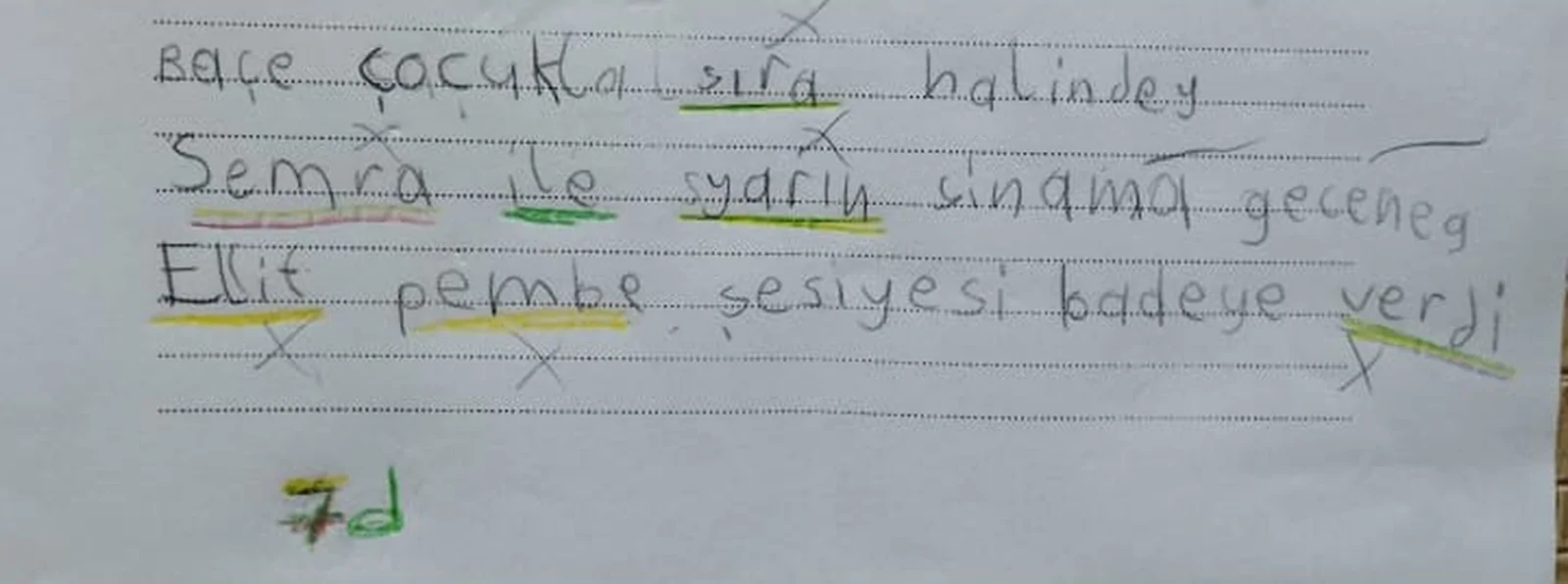
An example of a student’s writing style who can spell but struggles to write according to Turkish spelling.

For Example 3 (Fig 3), it is understood that the student recognized the Turkish letters but had not yet progressed to the stage of syllabification and conventional spelling. The student in Example 3 was, however, able to correctly write some words (three two-syllable words) which they had memorized.

**Fig 3:**
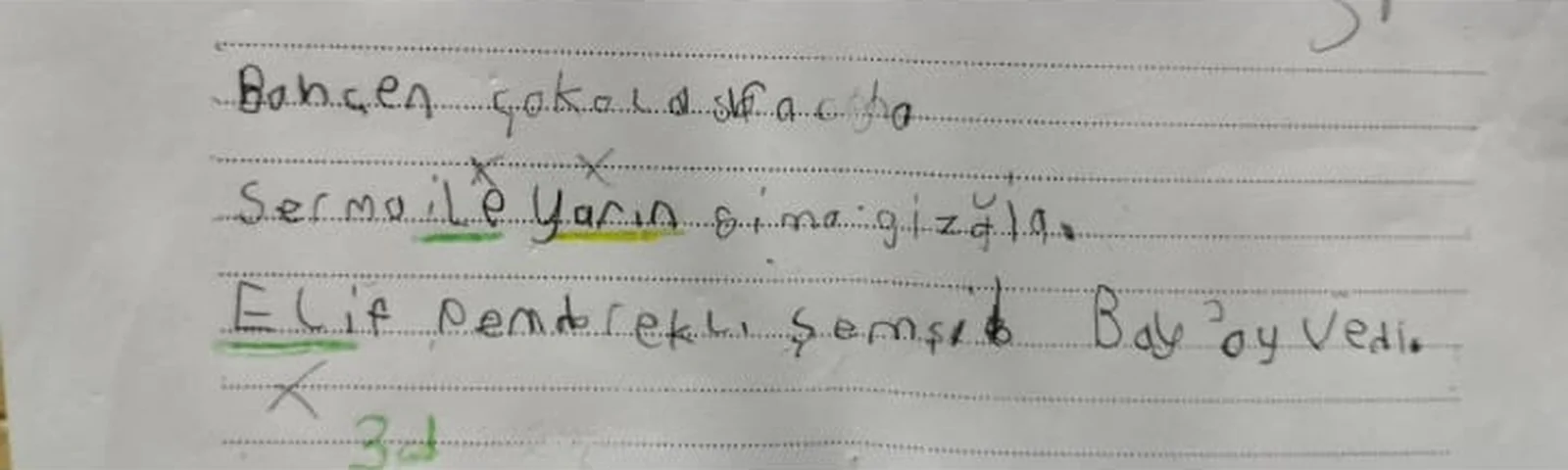
A writing sample of a student who is at the beginning of the spelling stage.

Each word written in compliance with Turkish orthographic conventions was scored as 1, whereas words that did not comply were scored as 0. Orthographic accuracy included correct grapheme usage and adherence to relevant spelling conventions (e.g., proper noun capitalization and correct suffixation, as in “Bade’ye”). The primary focus of the scoring procedure was the mechanical correctness of the written words. The objective of this approach was to determine whether students had successfully acquired the basic transcription skills required for written language. Punctuation knowledge was not included in the scoring criteria.

## Discussion

Within the correlational pattern observed, early writing performance was more strongly associated with phonological processing skills than with the isolated symbolic-production items of the LAP-3 (i.e., name and numeral writing), pointing to a central role for phonological awareness among the early skills examined here.

This longitudinal study indicates that preschoolers’ early writing performance in first grade is significantly and positively associated with their phonological awareness and emergent literacy skills. As the study relied on longitudinal correlational data rather than regression modeling with covariates, these associations are best interpreted as evidence consistent with, rather than direct confirmation of, well-established theoretical models that conceptualize writing as a developmental and multifaceted process shaped by early cognitive-linguistic abilities.

Theoretically, the results support several influential models of literacy development and our findings strongly align with established models of literacy development, particularly in underscoring the foundational role of phonological awareness (PA) and alphabetic principles in emergent writing skills.

The pattern of associations is consistent with H1 and H2: both PA and emergent literacy (LAP-3) were significantly and positively associated with early writing performance, and, consistent with H3, PA showed the numerically stronger association (r = .46 vs. r = .32). These results are broadly consistent with several influential accounts of early literacy development. Clay’s [27] emergent literacy perspective holds that letter recognition, phonological play, and early mark-making serve as precursors to conventional literacy from birth onward; our finding that PA and print-related skills were both associated with later writing is compatible with, though it does not by itself establish, this developmental account. Similarly, the association between PA—particularly syllable blending—and dictation performance is compatible with the Simple View of Writing [28], in which transcription (spelling and handwriting) is distinguished from text generation and executive functions; a dictation task of the kind used here is primarily a transcription measure, and PA is a plausible phonological substrate for transcription. The pattern is also compatible with Ehri’s model of word-reading development [29], in which syllable- and phoneme-level PA skills support the transition from logographic to alphabetic processing and, by extension, phonologically based spelling.

Because the present data are longitudinal correlations rather than regression estimates with covariates, they cannot adjudicate between these accounts or establish that PA causally drives transcription via reduced working-memory load, faster grapheme-phoneme mapping, or any other specific mechanism; such mechanistic claims would require experimental or fully covariate-adjusted designs. What the data support is a consistent, moderate-to-strong association between PA and early writing that is compatible with multiple complementary theoretical models rather than uniquely diagnostic of any one of them. Beyond the school-based skills examined here, home literacy practices such as dialogic reading are also known to shape children’s phonological awareness and vocabulary development [30], suggesting that family-level factors are a plausible complementary influence not captured by the present design.

This study extends the evidence base to a transparent orthography (Turkish), which has received comparatively little attention relative to English, a deep orthography with irregular grapheme-phoneme mappings. In our sample, PA remained significantly associated with writing performance even under highly consistent phoneme-grapheme mapping, a pattern broadly consistent with Sellés et al.’s [31] findings among Spanish-speaking children in Spain and Peru, where PA likewise emerged as a central correlate of early literacy in a transparent orthography. Rather than establishing a universal causal mechanism, this cross-linguistic consistency is more modestly interpreted as evidence that the association between PA and early literacy outcomes generalizes across at least some transparent orthographies, a question that future comparative and experimental work should test directly.

Name-writing ability showed a comparatively weak association with sentence-level writing skill in the present sample, despite being frequently treated as an emergent-literacy milestone [32]. This suggests that symbolic familiarity with one’s own name may be a limited proxy for the more analytical phonological and compositional skills that sentence-level writing requires, though a single weak correlation from one sample should not be over-interpreted.

Consistent with H4, neither writing performance, phonological awareness, nor emergent literacy differed significantly by age or gender. Several factors likely limited the power of these comparisons rather than indicating a true absence of any age- or gender-related effect. First, the sample spanned a narrow 66–77-month window, and age was analyzed using 2–3-month categorical bands (per Table 3); this fine-grained categorization, chosen to reflect the cohort structure in which children were originally enrolled, reduces statistical power relative to treating age as a continuous variable and should be considered a methodological limitation rather than a substantive finding of stability. Second, as reported in Table 5, the group-comparison analyses (Mann-Whitney U, Kruskal-Wallis) were themselves underpowered (1 − β = .30–.35) given the available sample size, further increasing the risk of Type II error. Consistent with cross-linguistic evidence that children acquire basic phoneme-grapheme correspondences relatively quickly in transparent orthographies such as Turkish [4], it remains plausible that foundational literacy skills were already converging within this age band by the time of assessment; however, this explanation is speculative and cannot be distinguished from a simple power limitation with the present design. Future studies should treat age as a continuous variable and recruit larger, age-heterogeneous samples to test this possibility directly, and should avoid over-interpreting the present non-significant age and gender comparisons as evidence of genuinely stable developmental profiles.

**Table 5:** Effect sizes and statistical power values for group comparisons and correlational analyses.

Analysis Type	Variables	Statistic	Effect Size	Effect Interpretation	Statistical Power (1 − β)	Power Interpretation
Mann–Whitney U	Gender – Writing	z = −1.054	r = 0.15	Small	0.30	Low
Mann–Whitney U	Gender – Phonological Awareness	z = −1.679	r = 0.17	Small	0.35	Low
Mann–Whitney U	Gender – Literacy	z = −1.696	r = 0.17	Small	0.35	Low
Spearman Correlation	Phonological – Writing	r = 0.46	r = 0.46	Medium	0.99	Very High
Spearman Correlation	Literacy – Writing	r = 0.32	r = 0.32	Medium	0.93	High
Spearman Correlation	Syllable Blending – Writing	r = 0.46	r = 0.46	Medium	0.99	Very High
Spearman Correlation	Syllable Segmentation – Writing	r = 0.43	r = 0.43	Medium	0.98	Very High
Kruskal–Wallis	Age – Writing	χ² = 2.26	η² ≈ 0.02	Very Small	0.07	Very Low

Effect sizes for Mann–Whitney U tests were calculated as r = *Z/ √N*; for Spearman correlations, r_s_ was utilized directly. All effect sizes were classified based on Cohen’s [21] benchmarks: small (r = .10), medium (r = .30), and large (r = .50). For Kruskal–Wallis tests, magnitude was assessed using eta squared (η²). Statistical power (1 − β) reflects the likelihood of detecting a true effect; values < .80 suggest inadequate power, necessitating a cautious interpretation of non-significant findings due to potential Type II errors.

The results indicate that while the correlational analyses achieved high statistical power, the group comparison tests yielded relatively low power levels, thereby increasing the risk of Type II errors. Consequently, non-significant differences between groups must be interpreted with caution. Conversely, the robust power observed in the correlational analyses reinforces the reliability of the identified associations—most notably the link between phonological awareness and writing. These findings likely represent meaningful effects rather than statistical artifacts. Nevertheless, further research utilizing larger cohorts is warranted to validate these observations.

Our findings are consistent with phonological awareness being an important correlate of early writing during the preschool period. Similar to the results reported by Wei et al. [33] in a Chinese sample, phonological processing skills are particularly influential in the early phases of literacy acquisition. However, their longitudinal data suggest that as children advance in grade level, other metalinguistic skills—such as morphological awareness—may become more predictive of literacy outcomes. This highlights the dynamic nature of literacy development and underscores the importance of age-appropriate interventions.

Although Turkish represents a highly transparent orthography with consistent grapheme–phoneme correspondence, PA was nonetheless significantly associated with early writing in this sample. This is consistent with, though it does not on its own establish, cross-linguistic generalizability of PA’s role in early writing; confirming genuine universality would require direct comparative or meta-analytic evidence across orthographies rather than a single-sample correlational study. With that caveat, the pattern observed here is compatible with instructional practices that prioritize phonological awareness skills regardless of orthographic transparency.

From a practical standpoint, the results emphasize the necessity of early intervention and structured literacy programs in preschool settings. Educators and policymakers should prioritize developmentally appropriate, play-based, and print-rich activities that enhance phoneme manipulation, syllable segmentation, and letter-sound knowledge. Integrating phonological awareness training into regular classroom routines can help ensure that children enter formal schooling with stronger foundational writing skills.

## Limitations

The most important limitation concerns analytic strategy. Although data were collected longitudinally, the associations reported here are based on Spearman rank-order correlations between variables measured at two time points rather than on regression models with covariates; correlational designs of this kind cannot establish prediction, causal mechanism, or developmental trajectory, and the findings throughout this manuscript are accordingly described as associations rather than as predictive or causal effects. Regression models incorporating relevant covariates (e.g., socioeconomic status, age as a continuous variable) would have allowed stronger inferential claims and are recommended for future studies using this or comparable designs.

Socioeconomic status (SES) was not measured or statistically controlled in this study, even though prior literature indicates that phonological awareness and early literacy skills vary with SES. This omission means that some of the observed associations between phonological awareness, emergent literacy, and writing performance could be partly confounded by unmeasured SES-related variation across the socioeconomically diverse district from which the sample was drawn. Future studies should collect and control for SES to isolate the unique contribution of phonological and emergent literacy skills.

As discussed above, age was treated as a categorical variable (2–3-month bands) rather than as a continuous variable, which reduces statistical power and may partly explain the non-significant age comparisons; this categorization reflected the cohort structure in which children were originally enrolled and assessed but is nonetheless a limitation of the analytic approach.

A notable limitation of the present study is the absence of a standardized measure of general cognitive ability (e.g., non-verbal IQ), which may have influenced the observed relationships among variables. Although efforts were made to ensure a developmentally typical sample—based on teacher reports and the exclusion of children with identified developmental difficulties—these procedures do not fully substitute for formal cognitive assessment. Therefore, it is possible that individual differences in general cognitive functioning contributed, at least in part, to variability in phonological awareness, emergent literacy, and early writing performance. This limitation is particularly relevant when interpreting non-significant group differences (e.g., age and gender), as insufficient control for cognitive ability may obscure subtle developmental effects. Future research should incorporate standardized cognitive measures to more precisely isolate the unique contributions of phonological awareness and emergent literacy to early writing development.

Additional limitations include the following: the visual-motor integration and early print-knowledge subdomains of the LAP-3 Early Literacy Subtest were not reported at the item level in Tables 1 and 4 (see Data Collection Tools); a formal statistical comparison of children retained versus those lost to follow-up between the two assessment waves was not conducted (see Study Group); and although the research sample approximates the target population, its limited representativeness of the broader population constitutes a further limitation. Future research should be conducted with larger and more diverse samples, particularly in urban contexts, to enhance the generalizability of the findings.

## Conclusion

In conclusion, this longitudinal study provides correlational evidence consistent with early writing development in preschoolers being a cognitively and linguistically complex process, related to foundational abilities such as phonological awareness and emergent literacy competencies rather than to motor maturation alone. The findings are consistent with established theoretical models of early literacy and extend their relevance to a transparent orthography, though genuine cross-linguistic universality would require direct comparative evidence beyond a single sample. Importantly, the comparatively limited association of isolated name-writing skill with sentence-level writing suggests a need to look beyond symbolic familiarity toward analytical phonological processing and early composition experiences. These findings carry plausible implications for educational practice, including the value of early, targeted interventions that foster phoneme awareness and letter knowledge to support writing acquisition; however, because the design was correlational, causal claims about the effectiveness of such interventions cannot be drawn from these data alone. Future research using regression-based or experimental designs should continue to explore these relationships across diverse orthographic systems and cultural contexts, while also investigating the roles of executive functioning and the home literacy environment.
